# Inhibitory effect of novel Eugenol Tosylate Congeners on pathogenicity of *Candida albicans*

**DOI:** 10.1186/s12906-020-02929-0

**Published:** 2020-04-29

**Authors:** Shabir Ahmad Lone, Aijaz Ahmad

**Affiliations:** 1 Clinical Microbiology and Infectious Diseases, School of Pathology, Health Sciences, University of the Witwatersrand, Johannesburg, 2193 South Africa; 2 Infection Control, Charlotte Maxeke Johannesburg Academic Hospital, National Health Laboratory Service, Johannesburg, 2193 South Africa

**Keywords:** *Candida albicans*, Eugenol tosylate congeners, Virulence factors, Biofilm

## Abstract

**Background:**

The global prevalence of fungal diseases is increasing rapidly, which affects more than a billion people every year with significant mortality rate. On the other hand, the development of new drugs to treat these fungal infections is slow, while the current antifungal therapy is insufficient and associated with adverse side effects and emerging multidrug resistance. Therefore, development of novel antifungal drugs with least or no toxicity and multi-target mechanisms of action is an immediate priority. Natural products have long been known to possess antimicrobial activities and are source of new drugs. Currently, modifying natural products to synthesize derivatives/analogues are of great scientific focus for discovering novel drugs with improved potency and safety. Modifications in eugenol to synthesize eugenol derivatives with enhanced antifungal activity have already been reported.

**Methods:**

In this study, three most active novel eugenol tosylate congeners (ETC-5, ETC-6 and ETC-7) were selected from our previous study to investigate their effect on major virulence factors of *Candida albicans* which include adherence, morphogenesis, hydrolytic enzymes secretion, biofilm formation and on expression of genes related to these virulence factors. Adherence and biofilm formation were studied by alamarBlue dye and XTT reduction assays respectively, hydrolytic enzyme secretion was evaluated by plate assays. Further, morphological transition was monitored microscopically and RT-qPCR was used to assess the gene expression levels.

**Results:**

ETCs significantly inhibited adherence in *C. albicans* with an inhibition range of 16–66%, and completely inhibited the morphogenesis at MIC values. Inhibition of proteinase and phospholipase activity was in the range of 2–48% and 8–34% respectively. Test compounds also significantly inhibit biofilm formation in *C. albicans* in the range of 7–77%. Furthermore, RT-qPCR results indicated a significant down regulation in expression levels of genes (*ALS1, ALS2, ALS3, ALS9, CPH1, HWP1, SAP1, SAP2, SAP3 and PLB1*) in *C. albicans* cells after treated with ETCs*.*

**Conclusion:**

The results indicated that these novel ETCs target major virulence factors of *C. albicans* and avert this commensal microbe to turn into pathogenic. However, further in-depth studies may facilitate the mechanisms involved by ETCs in targeting these virulence factors.

## Background

Candidiasis, caused by different *Candida* species is one of the most identified causes of hospital acquired infections with approximately 700,000 deaths worldwide annually [1–3]. *Candida* species are the 2nd most common causative agents of fungal infections worldwide and ranked 5th among hospital-acquired pathogens [4, 5]. However, 90% of invasive fungal infections due to *Candida* species are mostly attributed to *Candida albicans*, *Candida tropicalis, Candida glabrata*, *Candida krusei* and *Candida parapsilosis* [6]. Among these *C. albicans* remains the main causative agent [7]. *C. albicans* is an opportunistic pathogen, causing disease mainly in immunocompromised individuals and is associated with high morbidity and mortality rates [8, 9]. However, transition from the commensal to the pathogenic form involves several biochemical and morphological changes, which are known as virulence factors. The important virulence factors associated with *C. albicans* pathogenesis include adherence, morphogenesis, hydrolytic enzymes secretion and biofilm formation [10].

Adhesion to host cells is an important first step to initiate fungal infection. Following adherence, planktonic yeast form *C. albicans* cells transits to pathogenic hyphae forms leading to tissue invasion and biofilm formation [11–14]. Following hyphae formation, *C. albicans* cells secrete hydrolytic enzymes mainly proteinases and phospholipases, which are responsible for host tissue invasion [15]. With the increasing use of biomaterials, implants and use of medical catheters, *C. albicans* biofilm formation has drastically increased, which is directly correlated to increase in drug resistance. *C. albicans* biofilms are inherently resistant to most conventional antifungal drugs [16]. Moreover, currently available antifungal drugs are inadequate and constricted with limitations such as severe toxicity, narrow antifungal spectrum, emerging drug resistance and high costs [17, 18]. Thus, finding new antifungal drugs with alternative and multi-target mechanisms of action to develop more effective antifungal therapy has become an essential medical priority, especially to target multidrug resistant *Candida* species and resistant virulent forms such as biofilms.

Natural products are known for their antimicrobial activity; however, due to several limitations only few antifungals find their way to the clinics [19, 20]. Currently, modifying natural products to synthesize derivatives are of great interest to researchers for discovering novel molecules with improved potency and safety. Research in this area is promising for the development of new antifungal agents. Numerous studies have already reported that derivatization of eugenol enhanced its antifungal activity [19–23]. In our previous studies, we also reported that eugenol tosylate congeners (ETCs) possess potent antifungal activity by inhibiting the ergosterol biosynthesis pathway in *C. albicans* [19]. Based on our previous findings, we further modified eugenol to synthesis ETC with different functional groups and tested their antifungal activity against different *C. albicans* isolates [24]. In continuation to this study, we selected the three most active ETCs listed in Table 1 (ETC-5, ETC-6 and ETC-7) to explore their effect on major virulence factors of *C. albicans* in fluconazole susceptible and resistant *C. albicans* strains. The effect of these compounds on expression of genes related to pathogenicity of *C. albicans* has also been investigated.

**Table 1:** Eugenol tosylates congeners and their structures

Compound	Ligand name	Ligand structure	MIC^**a**^ (μg/ml)
**ETC-5**	4-allyl-2-methoxyphenyl 4-(trifluoromethyl) benzene		0.125–1.0
**ETC-6**	4-allyl-2-methoxyphenyl 4-(trifluoromethoxy) benzene		0.25–2.0
**ETC-7**	4-((4-allyl-2 methoxyphenoxy) sulfonyl) benzoic acid		1–8

^a^Minimum inhibitory concentrations are based on our previous findings [24]

## Methods

### Organisms and growth conditions

*C. albicans* strains including fluconazole susceptible *C. albicans* 4175, fluconazole resistant *C. albicans* 5112 and control strain *C. albicans* SC5314 (ATCC MYA- 2876) were used in this study. The clinical isolates were collected from patients visiting Charlotte Maxeke Johannesburg Academic Hospital, and for their presumptive identification germ tube assay and the API® 20 C AUX test kit (bioMérieux, France) were used. After identification all these isolates were stored in Sabouraud Dextrose broth (SDB) at − 80 °C supplemented with 20% glycerol in the Department of Clinical Microbiology and Infectious Diseases, University of the Witwatersrand, Johannesburg. The ethical clearance number M000402 obtained from the Human Research Ethics Committee, University of the Witwatersrand was used for these isolates. For experimental purposes each strain from the glycerol stock was recovered on Sabouraud Dextrose agar (SDA) plates at 37 °C for 24 h.

### Chemicals and drugs

All culture media and other chemicals used in this study were of high analytical grade. Sabouraud dextrose broth (Cat No-S3306), Sabouraud dextrose agar (Cat No-84088), RPMI-1640 Medium (Cat No-R6504), Phosphate buffered saline (Cat No-P4417), Fetal Bovine Serum (Cat No-F3885), Agar (Cat No-05040), Bovine Serum Albumin (Cat No-A9418), Yeast Nitrogen Base without Amino Acids (Cat No-Y0626), Ammonium sulphate (Cat No-A4418), Glucose (Cat No-G8270), Peptone (Cat No-77199), Sodium chloride (Cat No-S1679), Calcium chloride (Cat No-C1016), Egg Yolk Emulsion (Cat No-17148), MOPS (Cat No-M1254), XTT (Cat No-X4626), Menadione (Cat No-M5625), Acetone (Cat No-650501) were purchased from Sigma – Aldrich, St. Louis, MO, USA.

### Effect on virulence factors

The effect of three eugenol tosylate congeners at varying concentrations of ETC-5: 0.25 × MIC (0.032–0.25 μg/ml), 0.5 × MIC (0.063–0.5 μg/ml) and 1 × MIC (0.125–1 μg/ml), ETC-6: 0.25 × MIC (0.063–0.5 μg/ml), 0.5 × MIC (0.125–1 μg/ml) and 1 × MIC (0.25–2 μg/ml) and ETC-7: 0.25 × MIC (0.25–2 μg/ml), 0.5 × MIC (0.5–4 μg/ml) and 1 × MIC (1–8 μg/ml) on major virulence factors of *C. albicans* including adherence, morphogenesis, hydrolytic enzymes secretion, and biofilm formation have been studied, as these virulence factors can be utilised as emerging drug targets to develop new and effective antifungal drugs.

### Adherence assay

The adherence assay was performed using the technique described by Fazly et al.*,* 2013 [25]. Briefly, 100 μl of *C. albicans* cells (1 × 10^6^ cells/ml) grown in RPMI 1640 medium and buffered with 0.165 M Mops at pH 7.0 (Sigma – Aldrich, St. Louis, MO, USA) were inoculated into each well of Immulon 2HB 96-well microtiter plate. Cells were then exposed to different concentrations (0.25 × MIC, 0.5 × MIC and 1 × MIC) of ETC-5, ETC-6 and ETC-7 and incubated at 37 °C for 3 h. Untreated control cells were kept in every set of experiment as negative control. Subsequently incubation medium was removed and each well was washed two times with 200 μl PBS to remove non-adherent cells. A 100 μl of alamarBlue (Life Technologies) at a final concentration of 5% in RPMI 1640 medium were added to each well followed by incubation at 37 °C for 2 h. Fluorescence signals were read at 555_Ex_/585_Em_ using a BioTek Synergy HT microplate reader (BioTek Instruments, WA, USA).

### Morphogenesis

The effect of test compounds on yeast to hyphae transition was determined using a protocol described by Yousuf et al.*,* 2011 with modifications [26]. *C. albicans* cells were sub-cultured up to late log phase in SD Broth at 37 °C. Cells were then transferred into fresh SD broth followed by incubation at 37 °C for 48 h, for a synchronised cell population. Further, to induce hyphal formation 50 μl of synchronised cell population were transferred into 10 ml of fresh SD broth supplemented with 10% Fetal bovine serum (FBS) at pH 6.5. Varying concentrations of test compounds (0.25 × MIC, 0.5 × MIC and 1 × MIC) were added to the medium, followed by incubation at 37 °C. External pH of the medium was maintained at pH 6.5 and hyphae formation was observed microscopically using a Leica DM 500 microscope (Leica Microsystems, Heerbrugg Switzerland) by taking aliquots of 10 μl after 180 min. An untreated sample was also included as control.

### Proteinase assay

A proteinase assay was performed using a plate assay method described by Yousuf et al.*,* 2011 with modifications [26]. *C. albicans* cells were sub-cultured in 5 ml SD broth and incubated for 18 h at 37 °C. Cells were centrifuged at 3000 rpm for 5 min and resuspended in fresh medium. The cells were then exposed to desired concentrations of test compounds as mentioned above for 3 h. Cells without treatment served as negative control. Aliquots of 2 μl were placed at equidistant points on proteinase agar plates (agar 2%, BSA 2 g, yeast nitrogen base without amino acids, ammonium sulphate 1.45 g, glucose 20 g and distilled water 1000 ml). The plates were incubated for 3–4 days and then examined for proteinase activity by measuring clear zones of degradation using the formula:

$$ \mathrm{Pz}=\frac{\mathrm{diameter}\ \mathrm{of}\ \mathrm{colony}}{\mathrm{diameter}\ \mathrm{of}\ \mathrm{colony}+\mathrm{zone}\ \mathrm{of}\ \mathrm{degradation}} $$

### Phospholipase assay

*C. albicans* cells were exposed to different concentrations of test compounds for 3 h as described above. Aliquots of 2 μl were taken and placed on phospholipase agar plates (agar 2%; peptone 10 g; glucose 30 g; NaCl 57.3 g; CaCl_2_ 0.55 g and distilled water 900 ml enriched with 10% egg yolk emulsion) at equidistant points. The plates were incubated at 37 °C for 2–4 days and phospholipase activity was determined by measuring precipitation zones around the colonies [27]. Zones of precipitation were calculated as mentioned above.

### Biofilm formation

Biofilms were formed in flat-bottom 96-well microtiter plates by inoculating *C. albicans* cell suspensions (1 × 10^6^ cells/ml) in RPMI 1640 medium buffered with 0.165 M Mops pH 7.0 and incubated at 37 °C for 2 h [28]. After a 2 h adhesion period, the medium was removed carefully without disturbing the biofilm formation, and then varying concentrations of test compounds were prepared in fresh RPMI 1640 medium and added to the plate wells. The plates were further incubated at 37 °C for 24 h and 48 h. Effect of test compounds on biofilm formation were estimated using semi-quantitative XTT reduction assay.

### XTT reduction assay

A 2,3-Bis (2-methoxy-4-nitro-5-sulfo-phenyl)-2H-tetrazolium-5-carboxanilide (XTT) reduction assay was performed following the method described by Jin et al.*,* 2004 [29]. Briefly, XTT (1 mg/ml) in PBS and a solution of menadione (0.4 mM) in acetone was prepared. XTT – menadione solution was prepared fresh before each assay at a volume ratio of 20:1. The pre-formed biofilms were washed two times with PBS, then XTT (40 μl), menadione (2 μl) and PBS (158 μl) were added to each well of microtiter plates. The plates were incubated in the dark at 37 °C for 3 h. After incubation, the solution (100 μl) was transferred to each well of a new microtiter plate. The colorimetric changes were measured at 490 nm using iMark microplate reader (Bio-Rad laboratories, CA, USA). Reduction of XTT is directly correlated to the metabolic activity of biofilms.

### Gene expression analysis

Effect of ETCs on the expression of the genes for adherence (*ALS1, ALS2, ALS3* and *ALS9*), morphogenesis (*CPH1*, *HWP1)*, proteinases (*SAP1*, *SAP2,* and *SAP3*) and phospholipases (*PLB1)* was evaluated by RT-qPCR using method described by Ahmad et al.*,* 2015 with modifications [19]. Briefly, *C. albicans* cells at a concentration of 5 × 10^6^ cells/ml were exposed to the MIC of test compounds followed by incubation at 37 °C for 3 h. Untreated cells were used as negative control. Total RNA was extracted using Quick-RNA Fungal/Bacterial Miniprep Kit (Zymo Research, CA, USA) following the manufacturer’s instructions. Concentration of total RNA was measured using a Nanodrop 2000 spectrophotometer (Thermo Scientific, MA, USA). Thereafter, cDNA was synthesised using a iScript™ cDNA synthesis kit (Bio-Rad, CA, USA) as per manufacturer’s instructions. Primers for the above mentioned targets and housekeeping genes (*ACT1*, *PMA1* and *RPP2B*) were designed using NCBI/Primer3-Blast (https://www.ncbi.nlm.nih.gov/tools/primer-blast/) (Table 2) and synthesized from Integrated DNA Technologies, IA, USA. RT-qPCR was performed using PowerUp™ SYBR™ Green Master Mix (2X) (Thermo Fisher Scientific, MA, USA) in a RocheLight® Cycler Nano instrument Real-time PCR system (Roche, Basel, Switzerland). The following thermal cycling conditions for all RT-qPCR reactions were used; UDG activation at (50 °C/2 min), initial denaturation (95 °C/2 min), followed by 40 cycles of denaturation (95 °C/15 s), annealing (50 °C/60 s), and extension (72 °C/60 s). Melting curve analysis was performed to confirm the specificity of the primers. Furthermore, efficiency of the amplifications was confirmed by analysing the standard curves of both the target and housekeeping genes. Relative expression fold changes were assessed by ΔΔCT method using the formula 2^- (ΔΔCt).^

**Table 2:** List of primers used for RT-qPCR experiments

Gene	Primer	Sequence (5′–3′)	Amplicon length (bp)	Source
*ALS1*	Forward Reverse	*GCT CCA TCA CCT GCT GTT TC CTG AGG TGC CTG TTG TCA AG*	193	This study
*ALS2*	Forward Reverse	*TTT AAG GCT GGC ACC AAC AC ATT GTG AAC CCC ATT GCA CC*	203	This study
*ALS3*	Forward Reverse	*CTA CCG CTG TGA CCA CCT TA CAG TTT CCC CAA TTG GTG CA*	153	This study
*ALS9*	Forward Reverse	*CGA TTT CAG TTA GCA CCG CA CGT TGA AGT TGG CAC CTC TC*	242	This study
*CPH1*	Forward Reverse	*GTC GCC ACC CCA ACC TAT AT AGG AAA CCC AGA AGC GTC AT*	240	This study
*HWP1*	Forward Reverse	*CTG AAC CTT CCC CAG TTG CT CGA CAG CAC TAG ATT CCG GA*	174	This study
*SAP1*	Forward Reverse	*TTT GGT GGG GTT GAC AAA GC ATG ACC TTG ACC GTC CAG TT*	234	This study
*SAP2*	Forward Reverse	*CCG TTG GAT TTG GTG GTG TT AGC ATT ATC AAC CCC ACC GA*	239	This study
*SAP3*	Forward Reverse	*TGG TCC CCA AGG TGA AAT CA TGT CCT TGA CCA GCT TGA CA*	201	This study
*PLB1*	Forward Reverse	*ATA TGC TCC TGG TCC GGT TT ATT GCT CTA TAC CCT CCG CC*	231	This study
*ACT1*	Forward Reverse	*TGG TGA TGA AGC CCA ATC CA CAT TGG AGC TTC GGT CAA CA*	169	This study
*PMA1*	Forward Reverse	*GAA GGT GCT ACT GAT GCT GC GCA ACA TCA GCG AAA ATG GC*	242	This study
*RPP2B*	Forward Reverse	*ACA CCT CTC CAT CAG CTT CT TGG GAC AGA AGC TAA TTT GGT G*	155	This study

### Statistical analysis

Two-way ANOVA followed by Dunnett’s multiple comparisons test was used for statistical analysis, by using GraphPad Prism software, version 8.1.0. All the experiments were performed independently in triplicate (*n* = 3), and data were presented as mean ± standard deviation (SD). Values of p (****p < 0.0001, ***p **=** 0.0003, **p **=** 0.0045, *p **=** 0.0234) were considered as statistically significant.

## Results

### ETCs inhibit *Candida albicans* adhesion

**Fig. 1:**
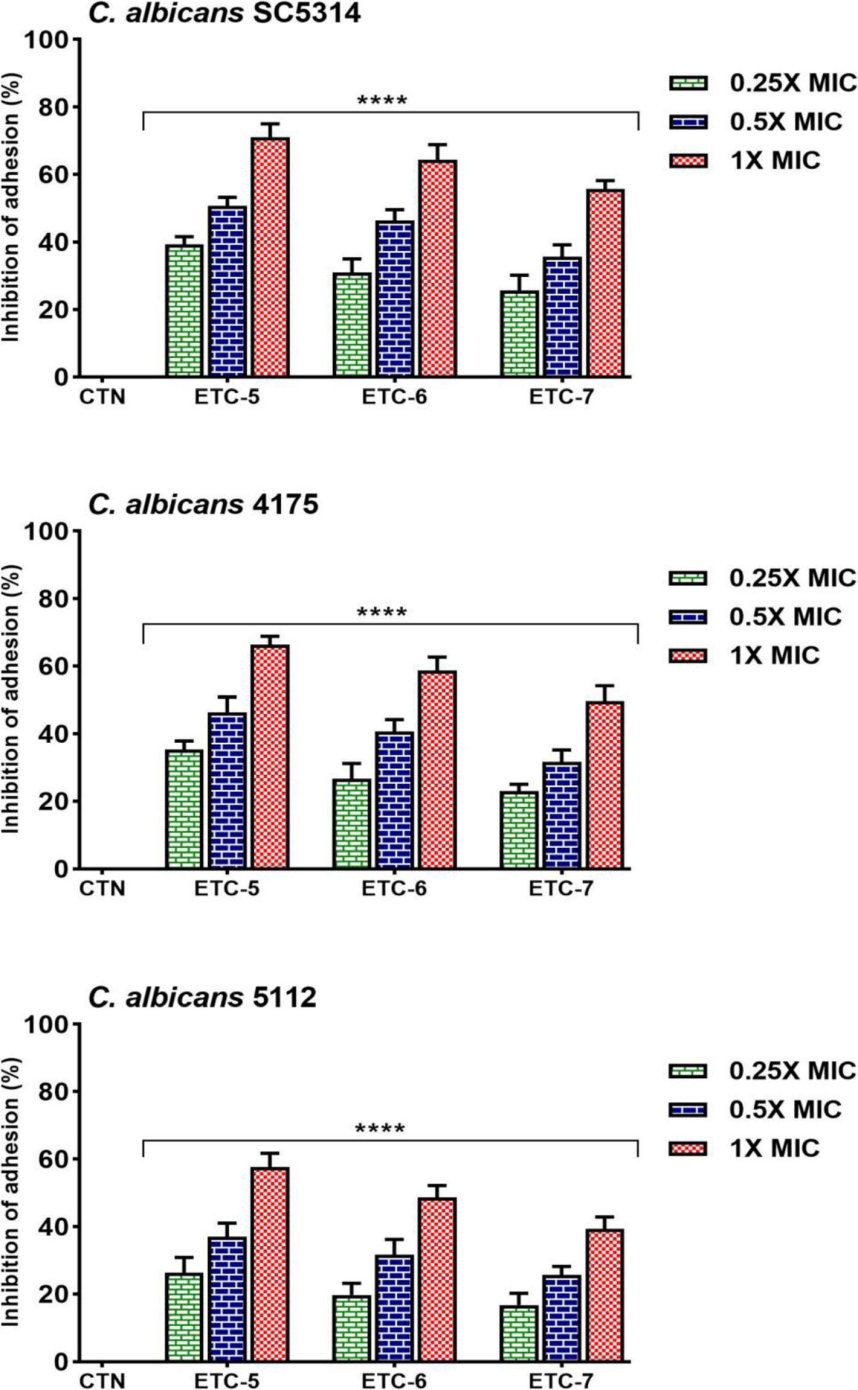
AlamarBlue-based polystyrene adhesion assay was used to evaluate the effect of test compounds on *C. albicans* adherence. Control *C. albicans* SC5314, fluconazole susceptible *C. albicans* 4175 and fluconazole resistant *C. albicans* 5112 strains were exposed to 0.25 × MIC, 0.5 × MIC and 1 × MIC values of test compounds for 3 h at 37 °C. Control bars (CTN) indicate untreated cells, accepted as 0% inhibition. Data are presented from three independent experiments using means ± S.D.****p < 0.0001

Adhesion assay using the vital dye alamarBlue as the detection reagent was used to evaluate the effect of ETC-5, ETC-6 and ETC-7 on *C. albicans* adherence. *C. albicans* cells after being exposed to different concentrations of test compounds exhibited varying degrees of inhibition of adherence, which indicates that the effect was concentration dependent (Fig. 1). For the control strain *C. albicans* SC5314 the test compounds were able to inhibit adhesion ranging from 55 to 71%, 35–50% and 25–39% at 1 × MIC, 0.5 × MIC and 0.25 × MIC respectively. The figures for fluconazole susceptible *C. albicans* 4175 and fluconazole resistant *C. albicans* 5112 strains were 49–66%, 31–46% and 23–35% and 39–57%, 25–37% and 16–26%, respectively. The results indicated that these test compounds were significantly effective in reducing *C. albicans* adhesion, even at sub-inhibitory concentrations.

### ETCs inhibit *Candida albicans* morphogenesis

**Fig. 2:**
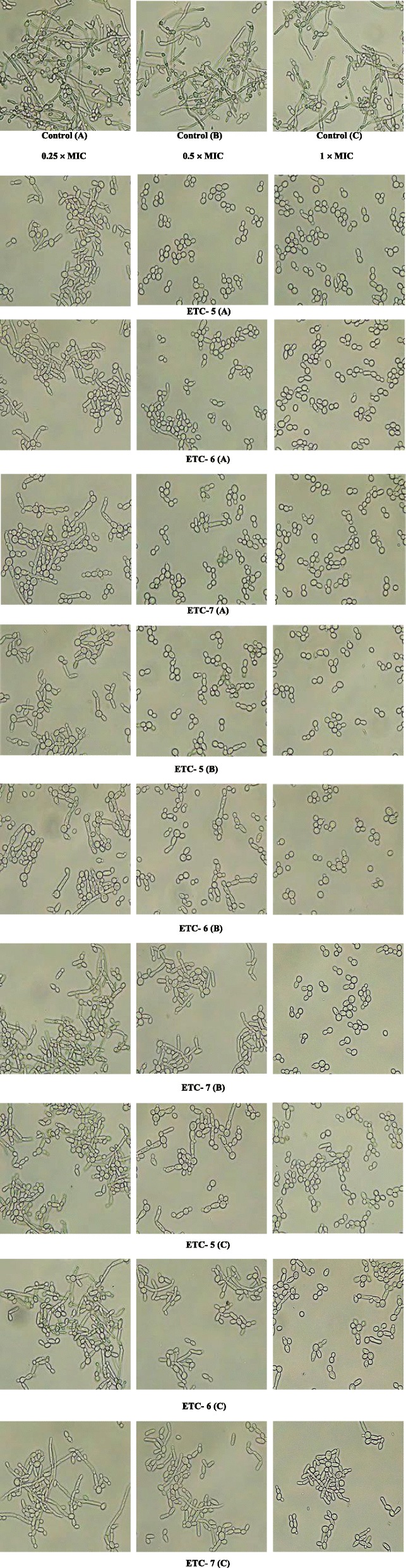
Yeast to hyphae transition in *Candida albicans* cells. *Candida* cells were grown in SD broth containing 10% FBS with 0.25 × MIC, 0.5 × MIC and 1 × MIC values of test compounds at 37 °C for 180 min. After incubation, an aliquot was taken from each sample and observed microscopically at 60X magnification. An untreated sample was used as control. The letters A, B and C represent control strain *C. albicans* SC5314, fluconazole susceptible strain *C. albicans* 4175 and fluconazole resistant strain *C. albicans* 5112 respectively

The effect of test compounds on yeast to hyphae transition in *C. albicans* cells were visualized microscopically. After exposure to different concentrations of test entities for 180 min, *C. albicans* cells showed significant dose dependent inhibition of hyphal growth when compared to their respective control cells, in which more than 90% of hyphal growth was observed after 180 min (Fig. 2). When compared to untreated control cells, complete inhibition of hyphal growth was observed in control *C. albicans* SC5314 and fluconazole susceptible *C. albicans* 4175 strains at 1 × MIC, followed by more than 90% inhibition at 0.5 × MIC values of test compounds. However, modest hyphal growth and shortened hyphae were observed in these *C. albicans* strains at 0.25 × MIC values of test compounds. Furthermore, against fluconazole resistant strain *C. albicans* 5112, hyphal growth was absent at 1 × MIC and modest at 0.5 × MIC. In contrast to fluconazole susceptible isolates, *C. albicans* 5112 showed hyphal growth at 0.25 × MIC, however not to the same degree as in control cells. All these findings demonstrated the potential of test compounds to inhibit transition of yeast cells to hyphae in both fluconazole susceptible and resistant *C. albicans* cells.

### ETCs inhibits *Candida albicans* hydrolytic enzyme secretion

We further studied the effect of eugenol tosylate congeners on secretion of hydrolytic enzymes (proteinases and phospholipases) in *C. albicans* cells. Results showed that *C. albicans* cells after being exposed to varying concentrations of test compounds inhibited enzyme secretion in a concentration dependent manner when compared to their respective untreated control cells.

**Fig. 3:**
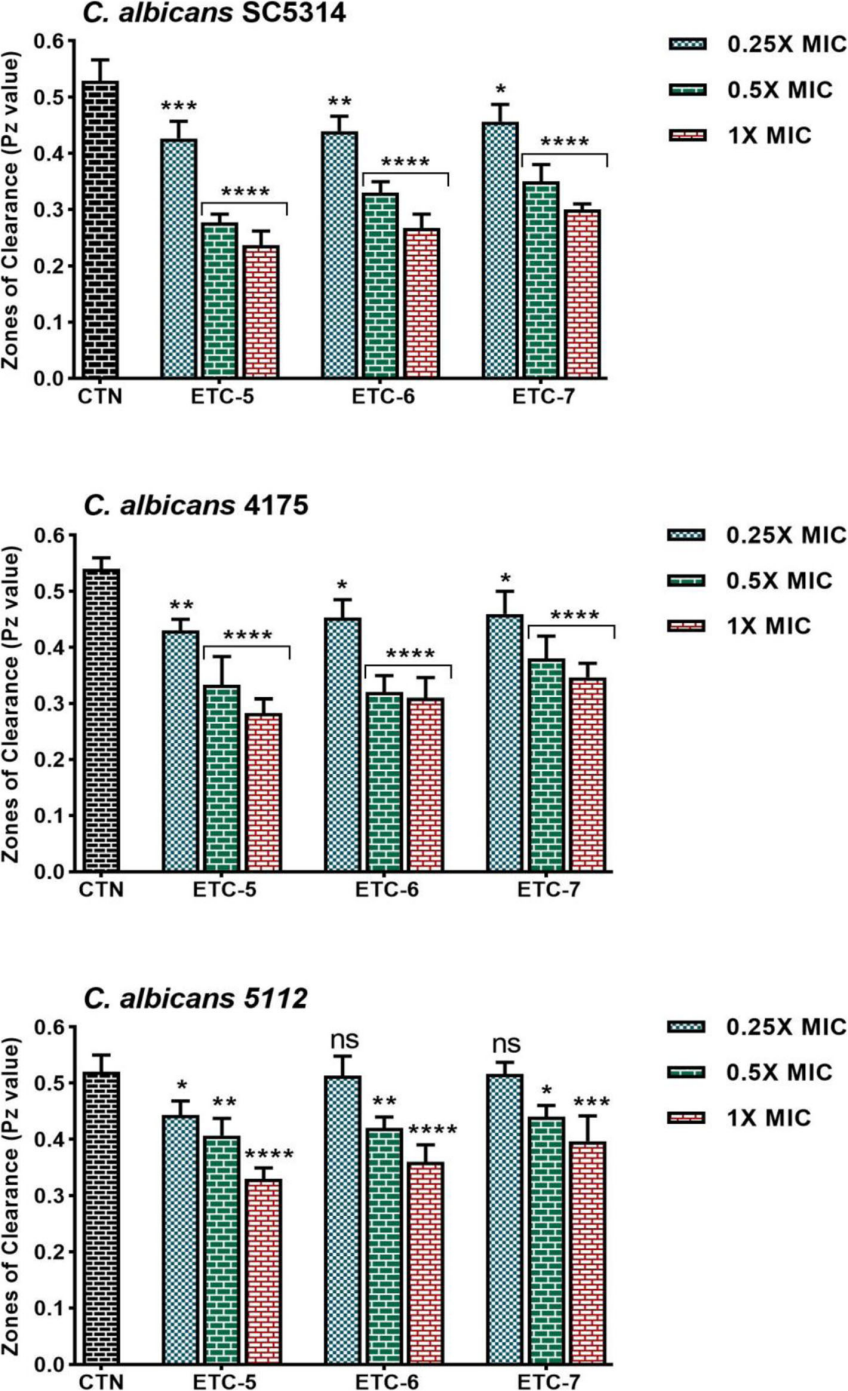
Effect of test compounds on proteinase secretion by *Candida albicans* strains. Control *C. albicans* SC5314, fluconazole susceptible *C. albicans* 4175 and fluconazole resistant *C. albicans* 5112 strains were exposed to 0.25 × MIC, 0.5 × MIC and 1 × MIC values of test compounds. Control bars (CTN) indicate untreated cells. High Pz value represents low enzyme activity. Data are presented from three independent experiments using means ± S.D.****p < 0.0001, ***p: 0.0003, **p: 0.0045, *p: 0.0234, ns: not significant

Figure 3 summarizes the proteinase activity of tested *C. albicans* strains. At 1 × MIC, test compounds exhibited 43–57%, 37–48% and 25–37% proteinase inhibition in control *C. albicans* SC5314, fluconazole susceptible *C. albicans* 4175 and fluconazole resistant *C. albicans* 5112 strains, respectively. At 0.5 × MIC and 0.25 × MIC values of test compounds proteinase inhibition in these *C. albicans* strains was observed in the range of 34–49%, 30–41%, 15–23% and 15–21%, 15–20%, 2–15%, respectively. Results demonstrated that test compounds significantly inhibited proteinase secretion in *Candida* cells. However, in fluconazole resistant *C. albicans* 5112 strain compounds, ETC-6 and ETC-7 did not have any significant effect on proteinase secretion at 0.25 × MIC value.

**Fig. 4:**
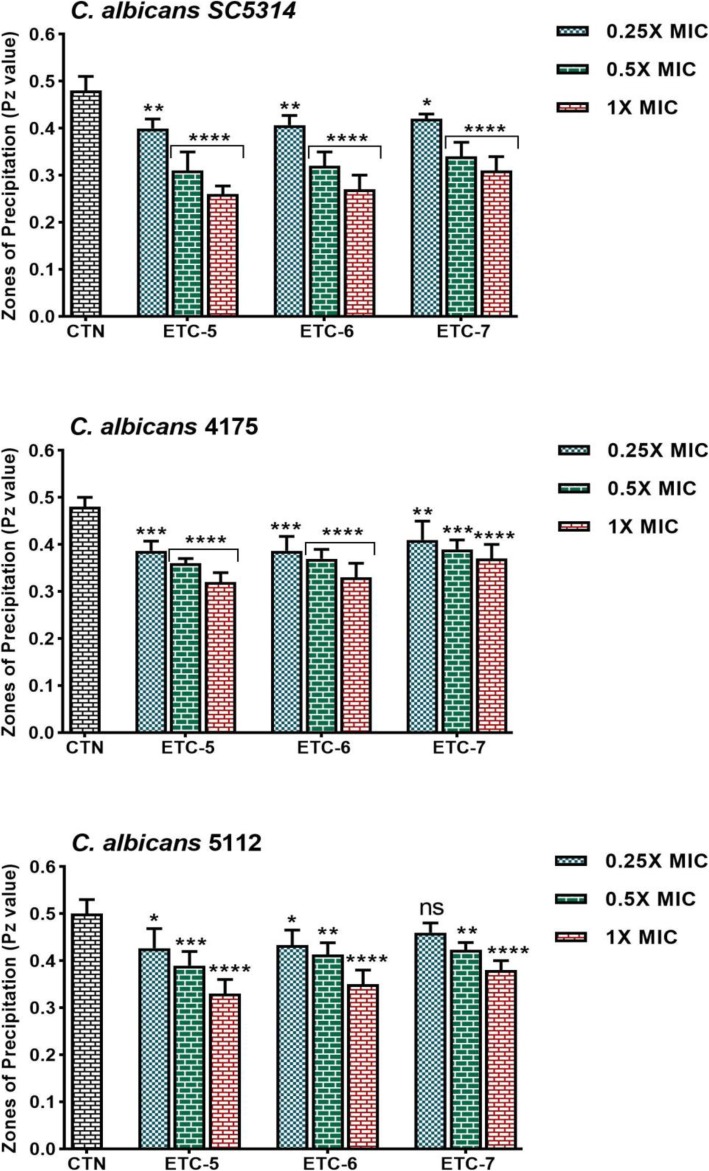
Effect of test compounds on phospholipase secretion by *Candida albicans* strains. Control *C. albicans* SC5314, fluconazole susceptible *C. albicans* 4175 and fluconazole resistant *C. albicans* 5112 strains were exposed to 0.25 × MIC, 0.5 × MIC and 1 × MIC values of test compounds. Control bars (CTN) indicate untreated cells. High Pz value represents low enzyme activity. Data are presented from three independent experiments using means ± S.D.****p < 0.0001, ***p: 0.0003, **p: 0.0045, *p: 0.0234, ns: not significant

Phospholipase enzyme activity results for all tested *C. albicans* strains are summarized in Fig. 4. Test compounds at 1 × MIC and 0.5 × MIC values showed significant inhibition in phospholipase secretion in the range of 35 to 46%, 23 to 33%, 24 to 34% and 29 to 35%, 19 to 25%, 16 to 22% in control *C. albicans* SC5314, fluconazole susceptible *C. albicans* 4175 and fluconazole resistant *C. albicans* 5112 strains, respectively. At 0.25 × MIC values, test compounds demonstrated significant inhibition in the phospholipase enzyme activity varying from 12 to 17% in standard strain, 15 to 21% in fluconazole susceptible strain and 8 to 16% fluconazole resistant strain of *C. albicans*. In fluconazole resistant *C. albicans* 5112 strain compound ETC-7 at 0.25 × MIC value did not significantly inhibit phospholipase enzyme secretion. These results indicated that test compounds decreased hydrolytic enzyme secretion in *C. albicans* cells to a varying extent.

### ETCs inhibits *Candida albicans* biofilm formation

**Fig. 5:**
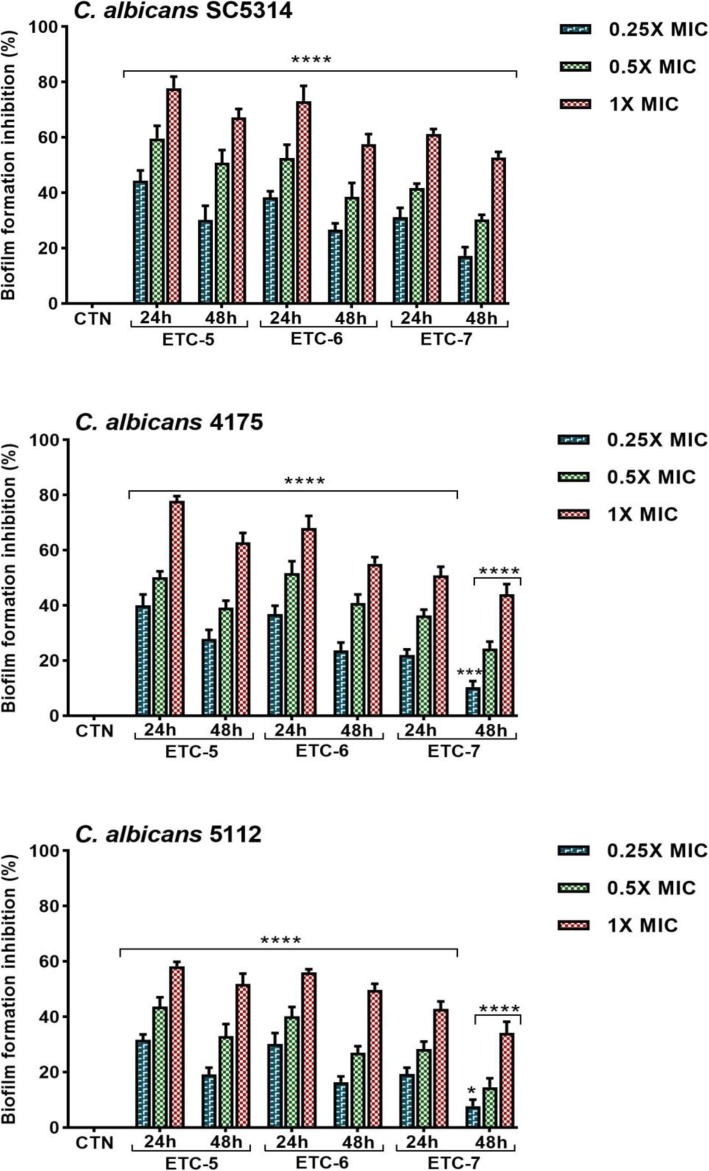
Effect of test compounds on *Candida albicans* biofilm formation. Control *C. albicans* SC5314, fluconazole susceptible *C. albicans* 4175 and fluconazole resistant *C. albicans* 5112 strains were incubated with 0.25 × MIC, 0.5 × MIC and 1 × MIC values of test compounds under biofilm-growing conditions for 24 and 48 h. Control bars (CTN) indicate untreated cells, accepted as 0% inhibition. Data are presented from three independent experiments using means ± S.D.****p < 0.0001, ***p: 0.0003, *p: 0.0234

Semi-quantitative XTT reduction assay was performed to observe the effect of test compounds on *C. albicans* biofilm formation. Significant inhibition in biofilm formation was observed in *C. albicans* cells after treatment with different concentrations of test compounds for 24 and 48 h (Fig. 5). At concentrations of 1 × MIC, 0.5 × MIC and 0.25 × MIC of test entities inhibition in biofilm formation in control strain *C. albicans* SC5314 was observed in the range of 52 to 77%, 30 to 59% and 17 to 44%, respectively. These ranges of percentage inhibition in biofilm formation in fluconazole susceptible *C. albicans* 4175 were found between 44 to 77%, 24 to 51% and 10 to 40%, respectively. Interestingly, significant inhibition in biofilm formation was also observed in fluconazole resistant *C. albicans* 5112 strain and the ranges were between 34 to 58%, 14 to 43% and 7 to 31% at 1 × MIC, 0.5 × MIC and 0.25 × MIC of test entities, respectively. Moreover, the rate of inhibition in biofilm formation by these test compounds was depended on concentration and treatment time. Our results showed that these ETCs drastically reduced biofilm formation in *C. albicans* cells.

### ETCs downregulates *Candida albicans* pathogenicity associated genes

**Fig. 6:**
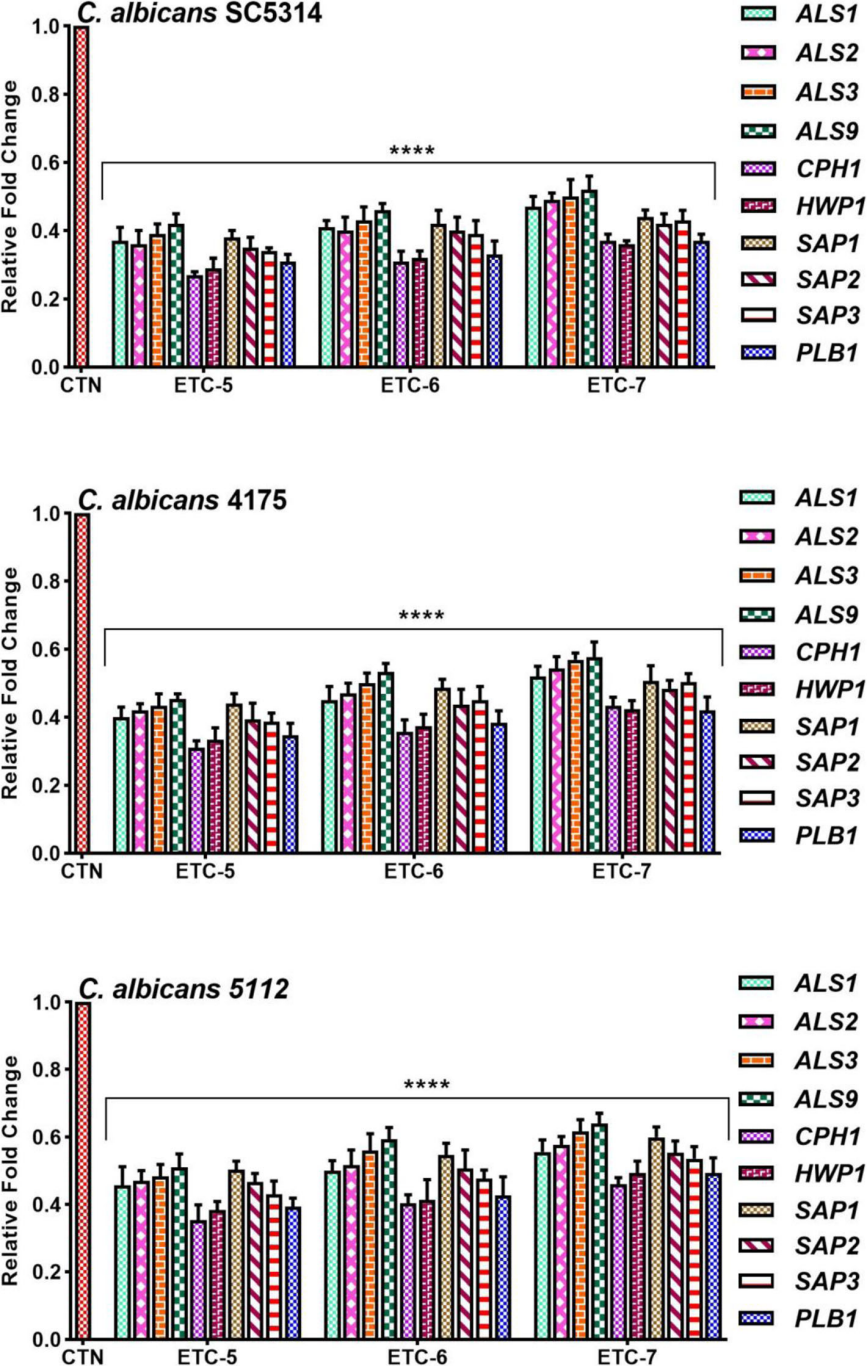
Relative expression of indicated genes after normalization to housekeeping genes (*ACT1*, *PMA1* and *RPP2B*) in control *C. albicans* SC5314, fluconazole susceptible *C. albicans* 4175 and fluconazole resistant *C. albicans* 5112 strains after exposure to test compounds (ETC-5, ETC-6 and ETC-7) at their respective MIC values. Cells without drug treatment (CTN) were used as negative control. Data are presented from three independent experiments using means of fold changes ± S.D.****p < 0.0001

Effect of test compounds (ETC-5, ETC-6 and ETC-7) on expression of important pathogenicity related genes (*ALS1, ALS2, ALS3, ALS9*, *CPH1*, *HWP1, SAP1*, *SAP2, SAP3* and *PLB1*) in *C. albicans* cells was tested and the results are summarized in Fig. 6. The expressions of the indicated genes are shown as relative values in comparison to the control (untreated cells) that were set to 1.0. Expression levels of all the indicated genes was down regulated significantly as compared to control cells when treated at MIC values of these test compounds. Expression of genes related to adherence (*ALS1, ALS2, ALS3* and *ALS9*), morphogenesis (*CPH1*, *HWP1)*, proteinases (*SAP1*, *SAP2,* and *SAP3*) and phospholipases (*PLB1*) in ETCs treated *C. albicans* SC5314 was reduced significantly with a fold range of 1.9 to 2.8, 2.7 to 3.7, 2.3 to 2.9 and 2.7 to 3.2 folds, respectively. These figures for fluconazole susceptible *C. albicans* 4175 and fluconazole resistant *C. albicans* 5112 were in the range of 1.8 to 2.5, 2.3 to 3.2, 2.0 to 2.6, 2.4 to 2.9 and 1.6 to 2.2, 2.0 to 2.9, 1.7 to 2.3, 2.0 to 2.6 folds, respectively. From these results it can be concluded that ETCs downregulated the expression of the above-mentioned genes, not only in fluconazole susceptible *C. albicans* but also in fluconazole resistant *C. albicans* cells.

## Discussion

Invasive fungal infections caused by different *Candida* species have increased considerably over the past few decades and has become a menace to global health. The unavailability of sufficient antifungal drugs and potential toxicity of currently available antifungals has restricted antifungal therapy. The development of new antifungal agents is restricted due to the limited number of known drug targets in fungi [20, 30]. These factors in turn amplify the emergence of resistance to the available antifungal agents and accentuate the need for development of novel antifungal drugs with different target sites. Based on these facts, we targeted major virulence factors in *C. albicans* such as adherence, morphological transition, secretion of aspartyl proteinase and phospholipases and biofilm formation by semi-synthetic ETCs. Several other studies have also reported antifungal activity of eugenol (parent compound for these tosylate congeners) and its derivatives [21–23]. Therefore, targeting virulence factors could be a new paradigm for the development of new and effective antifungal drugs with multiple drug targets.

*C. albicans* cells carry specialized proteins known as adhesins, which facilitate adherence to host cells and abiotic surfaces [31, 32]. In *C. albicans* agglutinin-like sequence (ALS) proteins (Als1 to Als7 and Als9) are well studied adhesins that encode cell surface glycoproteins. From the ALS family Als3 (hyphae associated) is particularly crucial for adhesion [33, 34]. From the results it was observed that the test compounds ETC-5, ETC-6 and ETC-7 significantly inhibited adherence in *C. albicans* in a dose-dependent manner with a range of 16–71%, and down regulated expression of adherence related genes (*ALS1, ALS2, ALS3* and *ALS9*) up to 1.9–2.8 folds. Furthermore, *C. albicans* adherence to host tissues and implanted medical devices leads to the formation of biofilms. Biofilm is one of the important virulence factor in *C. albicans*, which increases resistance to most conventional antifungal drugs [35]. Moreover, several studies have been reported that *C. albicans* biofilms are more resistant (up to a 1000 times) than planktonic cells to current antifungal therapy and it is assumed this resistance is due to the presence of matrix, which limits the penetration of these agents by creating a diffusion barrier [36–39]. Here, we found that ETCs drastically inhibited biofilm formation in a concentration dependent manner between 7 and 77% in both fluconazole susceptible and resistant *C. albicans* strains. We presume that ETCs target the membrane adhesin proteins which facilitate adherence thereby preventing adherence to host cells and abiotic surfaces, while subsequently inhibiting biofilm formation.

*C. albicans* has the capability of dimorphic switching (yeast to hyphae transition), one of its main virulence factor [13]. Hyphal forms of *C. albicans* not only play an essential role in biofilm formation [40], but are also important in facilitating *C. albicans* invasion into the host tissues thereby resulting in pathogenesis. Studies also reported that *C. albicans* in the hyphal form are more invasive than the yeast form [41]. Furthermore, various genes directly or indirectly are associated with hyphal formation which includes *HWP1*, *ALS1*, *ALS3* and *CPH1* [8, 42]. From the microscopic images it was revealed that ETCs at their MIC values completely inhibited hyphal growth and at 0.5 × MIC values more than 90% hyphae formation was inhibited. These results indicate that test compounds had a potent inhibitory effect on morphological transitions in *C. albicans* resulting in lack of hyphal growth. To understand the mechanism of ETCs in hyphal growth inhibition at molecular level, expression profile of genes related to hyphal growth were analyzed. Expression of *HWP1*, *CPH1, ALS1* and *ALS3* was reduced by 2.0–3.4 folds, 2.1–3.7 folds, 1.8–2.7 folds and 1.6–2.6 folds, respectively. These results demonstrated that the test compounds can potentially inhibit hyphae formation significantly by down regulating the expression of different hyphal genes. Previous studies have already reported a reduction in expression of the hyphae related genes resulting in inhibition of hyphal formation in *C. albicans* [8].

*C. albicans* is also capable of secreting extracellular hydrolytic enzymes, one of the important virulence traits that contribute in its pathogenicity. Secreted aspartyl proteinases (SAPs) and phospholipases (PLBs) are main enzymes which play an important role in adherence, invasion and damage of the host tissues [43]. Genes involved in secretion of hydrolytic enzymes mainly SAPs and PLBs are responsible for adherence, tissue damage, alteration in the host immune response and tissue invasion, disruption of host cell membrane, respectively [44, 45]. From the results, significant reduction in enzyme secretion was observed in cells after exposure to different concentrations of test compounds. This reduction amounted to between 2 and 57% and 8–46% for proteinases and phospholipases, respectively. Moreover, ETCs significantly down regulated expression of *SAP1*, *SAP2, SAP3* and *PLB1* genes by a fold range of 1.7–2.9 folds and 2.0–3.2 folds, respectively. The combined results from all the above assays exhibited a significant effect of ETCs on the major virulence factors in both fluconazole susceptible and resistant *C. albicans* strains.

This is a first study reporting the effect of eugenol derivatives on virulence factors of *C. albicans*; however, several studies have reported the effect of eugenol on virulence factors. A study by Raut and colleagues, showed eugenol significantly prevents morphogenesis, adhesion and biofilm development in *C. albicans* at MIC values of 0.031 mg/ml, 2 mg/ml and 0.5 mg/ml respectively [28]. In an another study, preformed biofilm of *C. albicans* treated with eugenol at 500 mg/L and 2000 mg/L for 48 h showed 50 and > 80% reduction in the metabolic activity of biofilms respectively [46]. Halbandge et al.*,* 2017 reported that eugenol at MIC value of 0.062 mg/ml inhibited invasive growth of *C. albicans* [47]. Our results are in agreement with these previous findings where eugenol was reported to have antifungal activity by targeting major virulence factors in *C. albicans*. However, when compared to these previous findings our results showed improved activity of these derivatives on virulence factors of *C. albicans* and proved that modifications in eugenol to synthesize eugenol derivatives not only enhanced the antifungal activity but also drastically decreased the MIC values of the compound. Results also revealed ETC-5 as the most active compound followed by ETC-6 and ETC-7, respectively.

## Conclusion

In conclusion, ETCs having potent antifungal activity also target major virulence factors of *C. albicans* and thereby inhibit the pathogenicity of this commensal microbe at the initial stages*.* These compounds significantly diminished *C. albicans* pathogenicity in terms of adherence, morphological transition, secretion of hydrolytic enzymes and biofilm formation both at biochemical as well as at molecular level. The in vitro results advocate further studies using animal models to reveal the in-depth mechanisms of these ETCs behind their antifungal activity, which may take these compounds to the next stage of drug development.

## Data Availability

Not applicable.

## Abbreviations

ETCs: Eugenol tosylate congeners
RT-qPCR: Quantitative reverse transcription PCR
ATCC: American type culture collection
SDB: Sabouraud Dextrose broth
SDA: Sabouraud Dextrose Agar
MIC: Minimum inhibitory concentration
FBS: Fetal bovine serum
BSA: Bovine Serum Albumin
NaCl: Sodium chloride
CaCl_2_: Calcium chloride
PBS: Phosphate-Buffered Saline
MOPS: (3-(N-morpholino) propanesulfonic acid)
API: Analytical profile index
ALS: Agglutinin-like sequence
SAPs: Secreted aspartyl proteinases
PLBs: Phospholipases
